# Ionic Liquid Aided [^11^C]CO Fixation for Synthesis of ^11^C‐carbonyls

**DOI:** 10.1002/open.202400410

**Published:** 2025-01-20

**Authors:** Anton Lindberg, Narges Mokhtarinori, Zhenzhen Yang, Sheng Dai, Ilja Popovs, Neil Vasdev

**Affiliations:** ^1^ Azrieli Centre for Neuro-Radiochemistry Brain Health Imaging Centre CAMH 250 College Street Toronto, ON M5T 1R8 Canada; ^2^ Chemical Sciences Division Oak Ridge National Laboratory Oak Ridge, TN USA; ^3^ Azrieli Centre for Neuro-Radiochemistry Brain Health Imaging Centre CAMH Department of Psychiatry University of Toronto 250 College Street Toronto, ON M5T 1R8 Canada

**Keywords:** Ionic liquid, Carbon-11, Radiochemistry, Positron emission tomography

## Abstract

Tributyl(ethyl)phosphonium oxopentenolate ([P_4442_][Pen]) is an ionic liquid developed to capture CO and has shown ability to catalyze carbonylation reactions in organic chemistry. Carbon‐11 (^11^C, t_1/2_=20.4 min) labeled CO is a highly versatile building block for the synthesis of positron emission tomography (PET) radiotracers that are applied for medical imaging. The use of [^11^C]CO is limited by its low solubility in organic solvents. Herein, we report a proof‐of‐concept study evaluating a new method to prepare ^11^C‐labeled amides, ureas and carbamates via reaction of [^11^C]CO in [P_4442_][Pen] and applied for fully automated radiosyntheses of Bruton's tyrosine kinase inhibitors, [^11^C]evobrutinib and [^11^C]ibrutinib.

## Graphical abstract

Tributyl(ethyl)phosphonium oxopentenolate ([P_4442_][Pen]) is an ionic liquid used in a proof‐of‐concept study evaluating its utility to trap carbon‐11 (t_1/2_=20.4 min)‐labeled CO for preparation of amides, ureas and carbamates.

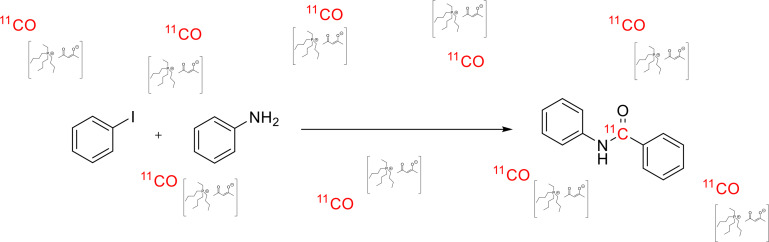

## Introduction

Positron emission tomography (PET) is a medical imaging technique commonly used in clinical trials and drug development to study biochemical interactions of radiotracers with receptors, enzymes or protein aggregates *in vivo*. Carbon‐11 (^11^C, t_1/2_=20.4 min) is a neutron deficient isotope of carbon which decays by emitting a positron. Incorporation of ^11^C into PET radiotracers is highly advantageous due to the presence of carbon in most biologically active compounds. Due to its short half‐life, the synthesis of ^11^C‐labeled compounds needs to be fast and efficient. Cyclotron produced ^11^C is generated via the ^14^N(p,α)^11^C nuclear reaction as either [^11^C]CO_2_ or [^11^C]CH_4_ and can be converted further into more reactive ^11^C‐labeled compounds such as [^11^C]CH_3_I or [^11^C]CH_3_OTf for ^11^C‐methylation reactions and [^11^C]CO_2_ can be used to prepare several other C1 building blocks such as [^11^C]CO for ^11^C‐carbonylation reactions.^1^, ^2^ [^11^C]CO is used for the synthesis of [^11^C]amides, [^11^C]esters/acids, [^11^C]carbamates and [^11^C]ketones.^2^, ^3^ However, [^11^C]CO is restricted by its relative low solubility in organic reaction solvents. One method to overcome this issue is utilizing palladium(0)/*Ni*‐Xantphos as a catalytic pair to serve as both a trapping agent and catalyst, and has been widely adopted for synthesis of [^11^C]amides.^4^, ^5^ Ionic liquids (ILs) are powerful solvents that can act as a phase‐transfer catalyst.^[6]^ IL's are used as a solvent/catalytic system for trapping of CO/CO_2_,^[7]^ but have limited applications in radiochemistry.^[8]^ We recently published on diazbicyclo[5.4.0]non‐5‐ene (DBN) and diazobicyclo[5.4.0]undec‐7‐ene (DBU) based ILs for the insertion of [^11^C]CO_2_ on aziridines.^[9]^ However, no such use of ILs has been reported for [^11^C]CO chemistry.

Tributyl(ethyl)phosphonium oxopentenolate ([P_4442_][Pen]) is an example of an IL developed for trapping atmospheric CO.^[10]^ [P_4442_][Pen] has demonstrated significantly enhanced CO capture through C‐site interaction. It achieves high CO absorption capacity (up to 0.03 mol CO per mol IL) under ambient conditions. The C‐site chemical interaction between the carbanion and CO has been confirmed through NMR and IR spectroscopic studies, as well as quantum mechanical computations. Additionally, it has shown promise as a catalyst for synthesizing *n*‐butyl benzoate under mild conditions with high yields, achieved by reacting *n*‐butanol with captured CO in [P_4442_][Pen].^[11]^ The ability to both capture and promote CO carbonylation reactions, makes [P_4442_][Pen] an attractive IL to implement in ^11^C‐carbonylation reactions for the synthesis of PET radiotracers.

**Scheme 1:**
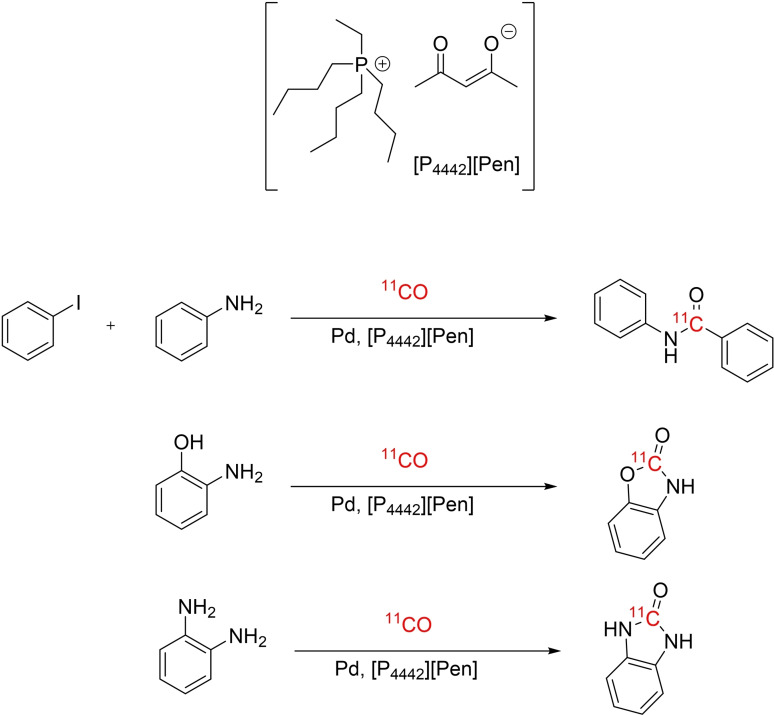
General scheme for carbonylation reaction using [P_4442_][Pen].

Herein, we present an initial proof‐of‐concept study on the application of [P_4442_][Pen] for ^11^C‐carbonylation reactions to prepare ^11^C‐amides from amines and aryl iodides, ^11^C‐carbamates from amines and alcohols and symmetrical ^11^C‐ureas from amines using [^11^C]CO (**Scheme** 1).

## Results and Discussion

Evaluation of [P_4442_][Pen] as a solvent/catalyst in [^11^C]CO carbonylation reactions were evaluated by measuring the trapping efficiency as the radioactivity in the reaction mixture after end of synthesis compared to the total amount of radioactivity added to the reaction vial. The application of [P_4442_][Pen] for radiolabeling of [^11^C]benzanilide with [^11^C]CO ([^11^C]**1**) was used as a starting point for reaction optimization (**Table** 1). Adapting the well‐utilized Pd(dba)_2_/*Ni*‐Xantphos method for ^11^C‐carbonylation via [^11^C]CO,^[4]^ the coupling of aniline and iodobenzene was performed with Pd(dba)_2_/[P_4442_][Pen] in *N*‐methyl‐2‐pyrrolidone (NMP) without the presence of *Ni*‐Xantphos in radiochemical conversion (RCC) of 30 % after trapping efficiency (TE) of 80 %. The role of the palladium catalyst was explored using a Pd(II) catalyst, Pd(PPh_3_)_2_Cl_2_, without any product being detected. Performing the radiolabeling without the [P_4442_][Pen] IL resulted in only 3 % TE and 2 % RCC.

**Table 1:** Optimization of [^11^C]benzanilide ([^11^C]**1**) radiolabeling using [P_4442_][Pen] and [^11^C]CO.

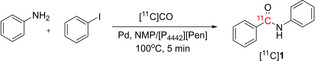				
Entry	Catalyst	Solvent	TE	RCC
1	Pd(dba)_2_	NMP/[P_4442_][Pen]	80 %	30 %
2	Pd(PPh_3_)_2_Cl_2_	NMP/[P_4442_][Pen]	30 %	‐
3	Pd(dba)_2_	NMP	3 %	2 %

*Reaction conditions*. Iodobenzene (20 μmol), aniline (20 μmol), Pd source (10 μmol) dissolved in NMP (100 μL), [P_4442_][Pen] (0 or 250 μL), and reacted with [^11^C]CO at 100 °C for 5 min. Trapping efficiency (TE), Radiochemical conversion (RCC).

The utility of [P_4442_][Pen] in [^11^C]CO carbonylation reactions was further evaluated in the radiolabeling of the urea functionality in [^11^C]benzoimidazolone ([^11^C]**2**) from the *ortho*‐substituted di‐amino precursor (**Table** 2). While the Pd(dba)_2_/[P_4442_][Pen] combination had high TE the RCC of [^11^C]**2** was only 6 %. Switching from the Pd(0) catalyst Pd(dba)_2_ to a Pd(II) catalyst, Pd(PPh_3_)_2_Cl_2_, afforded slightly higher RCC of 8 %. Addition of diisopropylethylamine (DIPEA) as a base resulted in an increased RCC of 23 %. As expected, attempts to perform the reaction without metal catalyst and with only the DIPEA base as reagent gave no product and low TE of 10 %.

**Table 2:** Optimization of [^11^C]benzoimidazolone ([^11^C]**2**) radiolabeling using [P_4442_][Pen] and [^11^C]CO.

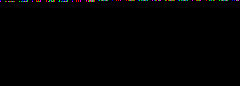				
Entry	Catalyst	Solvent	TE	RCC
1	Pd(dba)_2_	NMP/[P_4442_][Pen]	90 %	6 %
2	Pd(PPh_3_)_2_Cl_2_	NMP/[P_4442_][Pen]	85 %	8 %
3	Pd(PPh_3_)_2_Cl_2_, DIPEA	NMP/[P_4442_][Pen]	70 %	23 %
4	DIPEA	NMP/[P_4442_][Pen]	10 %	‐

*Reaction conditions*. 1,2‐diaminobenzene (20 μmol), Pd source (10 μmol) dissolved in NMP (100 μL), [P_4442_][Pen] (0 or 250 μL), DIPEA (0 or 8 μL) 100 °C for 5 min.

Another application of [P_4442_][Pen] investigated was the [^11^C]CO carbonylation of the carbamate functionality in [^11^C]benzoxazolone ([^11^C]**3**) from the respective amino alcohol precursor (**Table** 3). Using Pd(dba)_2_ as the catalyst with [P_4442_][Pen] resulted in high TE of 90 % but only 6 % RCC. Addition of DIPEA increased the RCC slightly to 8 %. Increasing the reaction temperature to 140 °C and the reaction time to 10 min did not result in any improvements to either TE or RCC. Changing the catalyst to Pd(PPh_3_)_2_Cl_2_ increased the RCC to 38 %, even though the TE was lower at 70 %. The control reaction condition without [P_4442_][PEN] resulted in drastically lower TE of 5 % with RCC of 3 %.

**Table 3:** Optimization of [^11^C]benzoxazolone ([^11^C]**3**) radiolabeling using [P_4442_][Pen] and [^11^C]CO.

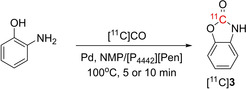				
Entry	Catalyst	Solvent	TE	RCC
1	Pd(dba)_2_	NMP/[P_4442_][Pen]	90 %	6 %
2	None	NMP/[P_4442_][Pen]	60 %	‐
3	Pd(dba)_2_, DIPEA	NMP/[P_4442_][Pen]	90 %	8 %
4^[a]^	Pd(dba)_2_, DIPEA	NMP/[P_4442_][Pen]	70 %	4 %
5	Pd(PPh_3_)_2_Cl_2_, DIPEA	NMP/[P_4442_][Pen]	70 %	38 %
6	Pd(PPh_3_)_2_Cl_2_, DIPEA	NMP	5 %	3 %

*Reaction conditions*. 2‐aminophenol (20 μmol), Pd source (0 or 10 μmol) dissolved in NMP (100 μL), [P_4442_][Pen] (0 or 250 μL), DIPEA (0 or 8 μL) 100 °C for 5 min. [a]140 °C for 10 min.

**Figure 1:**
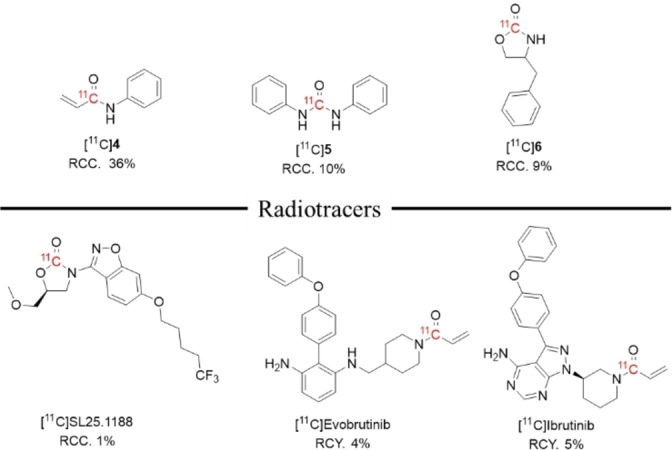
PET radiotracers labeled with [^11^C]CO using [P_4442_][Pen] as the solvent.

Based on the results from the preliminary evaluations of [P_4442_][Pen] in [^11^C]CO radiolabeling reactions, an array of labeled compounds consisting of [^11^C]phenylacrylamide ([^11^C]**4**), [^11^C]diphenylurea ([^11^C]**5**) and [^11^C]benzyloxazolidinone ([^11^C]**6**) was explored using the optimized conditions for ^11^C‐labeled amides, ureas and carbamates, respectively (**Figure** 1). Additionally, three PET radiotracers, [^11^C]SL25.1188, a ^11^C‐oxazolidinone for imaging MAO−B,^[12]^ as well as two ^11^C‐*N*‐acrylamide radiotracers for imaging Bruton's tyrosine kinase, [^11^C]evobrutinib^[13]^ and [^11^C]ibrutinib^[14]^ were selected for fully automated labeling using the optimized conditions for carbamates and amides (**Figure** 1).

The optimal conditions from the radiolabeling of [^11^C]**1** were applied to the radiolabeling of [^11^C]**4** in RCC of 36 %. The optimized reaction conditions for [^11^C]**2** were applied to the synthesis of the symmetrical [^11^C]diphenylurea ([^11^C]**5**) in RCC of 10 %, and the conditions for [^11^C]**3** were applied to the synthesis of [^11^C]benzyloxazolidinone ([^11^C]**6**) in RCC of 9 %.

With the successful labeling of the ^11^C‐oxazolidinone moiety, the method was automated to radiolabel [^11^C]SL25.1188 and resulted in RCC of 1 %, and is much lower than our method to routinely synthesize this radiopharmaceutical by [^11^C]CO_2_‐fixation.^[15]^ Due to the low RCC, [^11^C]SL25.1188 could not be isolated. However, the optimized conditions for [^11^C]**4**, were successfully translated for radiolabelling of [^11^C]ibrutinib and [^11^C]evobrutinib at the *N*‐^11^C‐acrylamide moiety in clinically relevant quantities. The fully automated syntheses yielded [^11^C]Ibrutinib in RCY of 5 % with molar activity (A_m_) of 27.3 GBq/μmol and [^11^C]evobrutinib in RCY of 4 % an A_m_ of 25.3 GBq/μmol. Both tracers were isolated using semi‐preparative HPLC according to our previously published methods.^13^, ^14^ The IL eluted with the void volume on HPLC and could not be detected in the isolated product by further HPLC analysis. The RCY achieved for the radiolabeling of [^11^C]ibrutinib and [^11^C]evobrutinib with [P_4442_][Pen] are comparable to the RCYs achieved by reactions with [^11^C]CO using Pd(dba)_2_/*Ni*‐Xantphos.^13^, ^14^

This preliminary study shows that [P_4442_][Pen] can be combined with both Pd(0) and Pd(II) in ^11^C‐carbonylation reactions with [^11^C]CO as a proof‐of‐concept. Additionally, three PET radiotracers were successfully radiolabeled in a fully automated manner, namely, [^11^C]SL25.1188, [^11^C]ibrutinib and [^11^C]evobrutinib, of which the latter two were isolated.

## Conclusions

[P_4442_][Pen] is shown to be a useful IL for [^11^C]CO radiochemistry, with applications for the synthesis of ^11^C‐amides, ^11^C‐ureas and ^11^C‐carbamates and was successfully applied to the fully automated radiosynthesis of [^11^C]SL25.1188, [^11^C]ibrutinib and [^11^C]evobrutinib. Further use of [P_4442_][Pen] and other CO/CO_2_ binding ILs represent an alternative labeling strategy for [^11^C]CO/[^11^C]CO_2_ radiochemistry.

## Experimental Section

[P_4442_][Pen] IL was synthesized by neutralizing acetylacetone with phosphonium hydroxide. In a typical experiment, an ethanol solution of tributyl(ethyl)phosphonium hydroxide ([P_4442_]OH) was prepared from [P_4442_]Br using the anion‐exchange resin method as described in previous publications.^10^, ^16^ An equimolar amount of acetylacetone was added to the ethanol solution of [P_4442_]OH. The mixture was then stirred at room temperature overnight. The produced IL was then vacuum‐dried for 48 hours at 80 °C to remove the solvent. The structure of the IL was confirmed by ^1^H and ^13^C NMR spectroscopy.

No‐carrier added [^11^C]CO_2_ was delivered to a TracerMaker synthesis platform (Scansys Laboratorieteknik, Denmark). [^11^C]CO was generated by passing [^11^C]CO_2_ in helium carrier gas through a molybdenum oven heated to 850 °C and trapped on molecular sieves cooled with liquid nitrogen. [^11^C]CO was delivered to a reaction vial and bubbled through the reaction mixture. After the reaction the head space in the reaction vial was vented to replace the gas in the headspace of the reaction vial before measuring radioactivity.

Optimized radiochemistry procedure. [^11^C]amides were synthesized starting from the appropriate iodide and amine (20 μmol) with Pd(dba)_2_ (3 mg). NMP (100 μL) was added to dissolve the reagents before [P_4442_][Pen] (250 μL) was added. [^11^C]CO was bubbled through the reaction mixture. Once maximum radioactivity was reached, the inlet and outlet line were removed and the reaction vial was heated for 5 min. [^11^C]Ureas and carbamates were synthesized from the appropriate amines or amino alcohols (50 μmol) with Pd(PPh_3_)_2_Cl_2_ (3 mg) and DIPEA (8 μL). NMP (100 μL) was added to dissolve the reagents before [P_4442_][Pen] (250 μL) was added. [^11^C]CO was bubbled through the reaction mixture. Once maximum radioactivity was reached, the inlet and outlet line were removed and the reaction vial was heated for 5 min. TE was measured as the percentage of radioactivity left in the reaction mixture at end of synthesis after the head space of the reaction vial had been flushed compared to the total radioactivity delivered to the reaction vial. RCC was measured by radio‐HPLC as the percentage of desired radiochemical product in the reaction mixture at end of synthesis, taking into account TE as well. Non‐decay corrected RCY was measured as the radioactivity of an isolated sample of the desired radiochemical product compared to the radioactivity of [^11^C]CO at the start of synthesis. A_m_ was calculated by comparing the area under the curve of the radiolabeled product with a reference sample of known concentration.

## Conflict of Interests

The authors declare no conflict of interest.

## Supporting information

As a service to our authors and readers, this journal provides supporting information supplied by the authors. Such materials are peer reviewed and may be re‐organized for online delivery, but are not copy‐edited or typeset. Technical support issues arising from supporting information (other than missing files) should be addressed to the authors.

Supporting Information

## Data Availability

The data that support the findings of this study are available from the corresponding author upon reasonable request.
