# Supplementary material for: Ionic Liquid Aided [11C]CO Fixation for Synthesis of 11C‐carbonyls
Source: ChemistryOpen. 2025 Jan 20;14(7):e202400410. doi: 10.1002/open.202400410 (PMC13042876; doi:10.1002/open.202400410)
Supplement: Supplementary file 1 — Supporting Information [file OPEN-14-e202400410-s001.pdf]

ChemistryOpen

Supporting Information

Ionic Liquid Aided [^{11}C]CO Fixation for Synthesis of ^{11}C-carbonyls

Anton Lindberg,* Narges Mokhtarinori, Zhenzhen Yang, Sheng Dai, Ilja Popovs, and Neil Vasdev*

Supporting information

Ionic Liquid aided [^{11}C]CO fixation for synthesis of ^{11}C-carbonyls

Anton Lindberg,[a]* Zhenzhen Yang,[b] Narges Mokhatarinori,[c] Sheng Dai,[d] Ilja Popovs,[e] and Neil Vasdev[f]*

[a] Dr. Anton Lindberg
Azrieli Centre for Neuro-Radiochemistry, Brain Health Imaging Centre, CAMH, Canada
250 College Street, Toronto, ON M5T 1R8
E-mail: anton.lindberg@camh.ca

[b] Narges Mokhtarinori
Chemical Sciences Division, Oak Ridge National Laboratory
Oak Ridge, TN, USA

[c] Dr. Zhenzhen Yang
Chemical Sciences Division, Oak Ridge National Laboratory
Oak Ridge, TN, USA

[d] Dr. Sheng Dai
Chemical Sciences Division, Oak Ridge National Laboratory
Oak Ridge, TN, USA

[e] Dr. Ilja Popovs
Chemical Sciences Division, Oak Ridge National Laboratory
Oak Ridge, TN, USA

[f] Professor Neil Vasdev
Azrieli Centre for Neuro-Radiochemistry, Brain Health Imaging Centre, CAMH, Canada
Department of Psychiatry, University of Toronto, Canada
250 College Street, Toronto, ON M5T 1R8

Table of contents:

^{1}H and ^{13}C NMR spectra for [P_{4442}][Pen] 2-3

HPLC Radiochromatograms 4-14

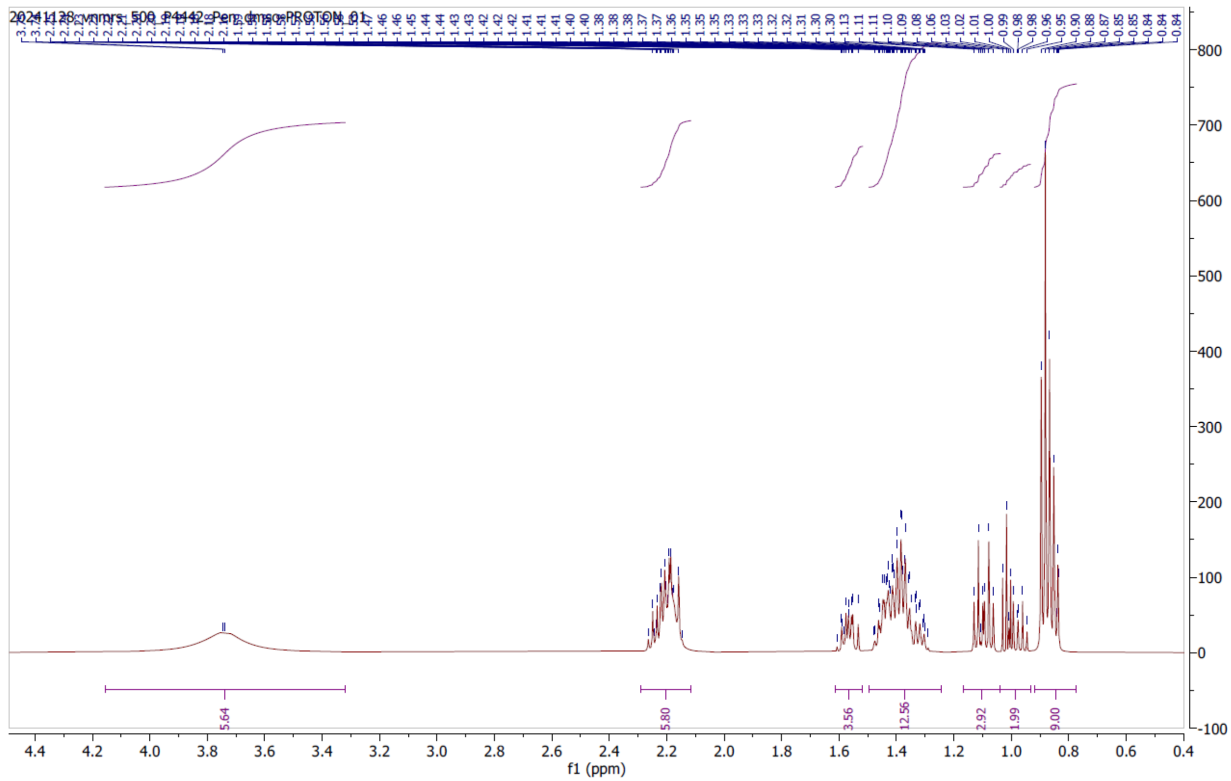

Figure S1. ^{1}H NMR (500 MHz, DMSO-d6) spectrum of the [P_{4442}][Pen). (DMSO-d_6):^{1}H NMR (500 MHz, DMSO-d_6) δ 4.16 – 3.32 (m, 6H), 2.21 (tdd, *J* = 16.6, 13.5, 8.2 Hz, 6H), 1.61 – 1.52 (m, 3H), 1.50 – 1.24 (m, 12H), 1.17 – 1.04 (m, 3H), 1.04 – 0.93 (m, 2H), 0.92 – 0.77 (m, 9H).

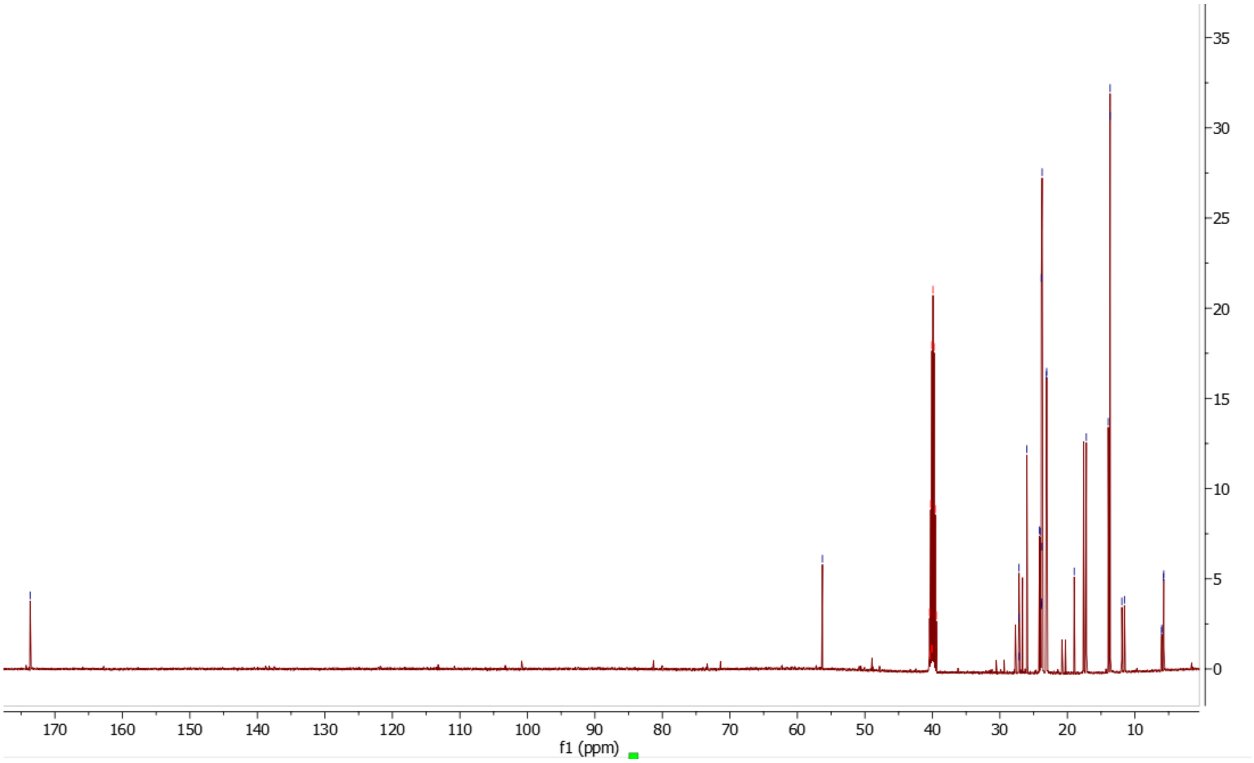

Figure S2. ^{13}C NMR (126 MHz, DMSO-d$_6$) spectrum of the [P$_{4442}$][Pen]. ^{13}C NMR (126 MHz, DMSO-d$_6$) δ 173.67, 56.28, 27.19, 27.16, 27.14, 25.99, 24.13, 24.02, 23.86, 23.82, 23.79, 23.77, 23.74, 23.10, 23.07, 18.98, 17.19, 13.95, 13.67, 13.66, 11.92, 11.54, 6.05, 6.01, 5.76, 5.72.

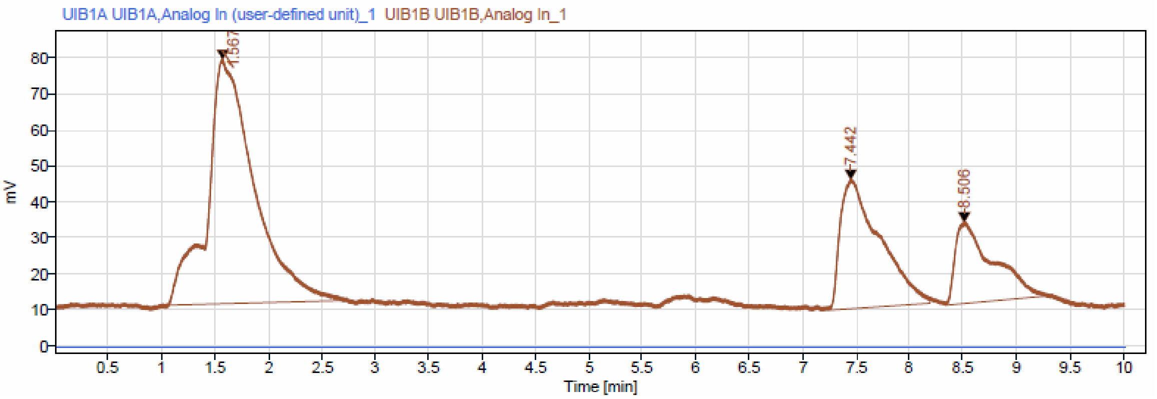

Figure S3. Radio-HPLC chromatogram of reaction mixture with optimized conditions for [^{11}C]**1**. Retention time for reference standard of benzanilide (**1**) = 7.35 min.

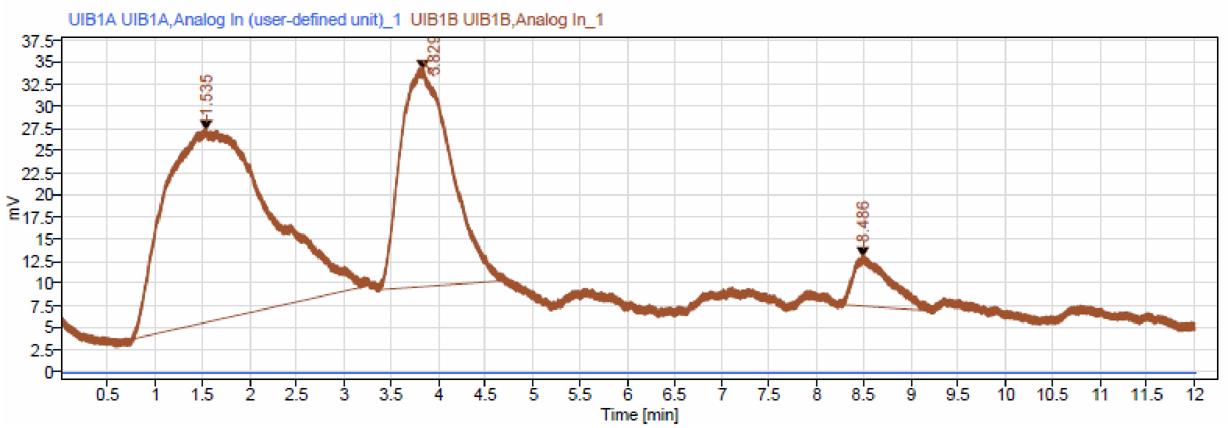

Figure S4. Radio-HPLC chromatogram of reaction mixture with optimized conditions for [^{11}C]**2**. Retention time for reference standard of benzoimidazolone (**2**) = 3.55 min.

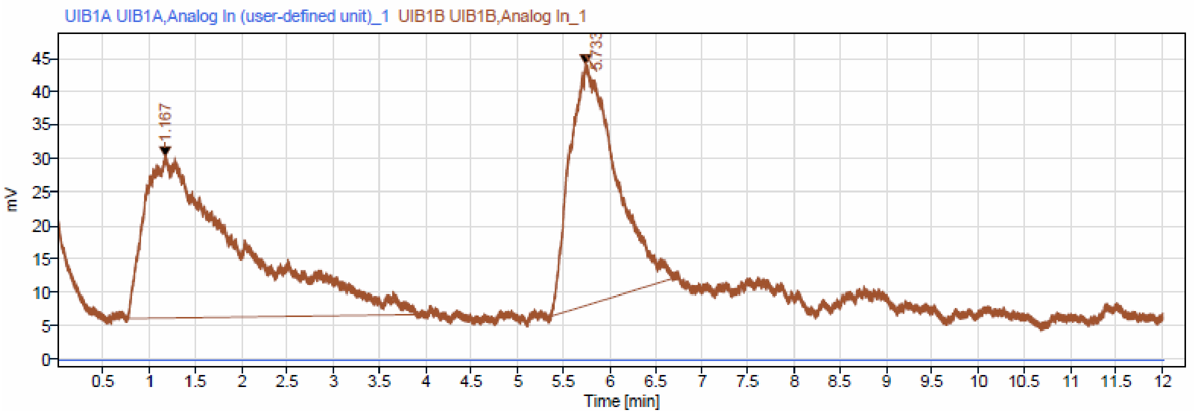

Figure S5. Radio-HPLC chromatogram of reaction mixture with optimized conditions for [^{11}C]**3**. Retention time for reference standard of benzoxazolone (**3**) = 5.55 min.

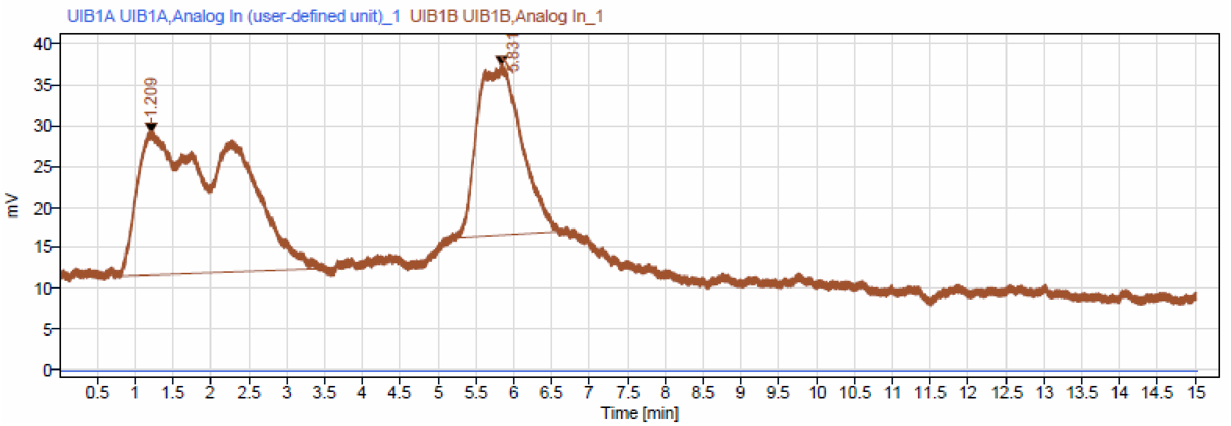

Figure S6. Radio-HPLC chromatogram of reaction mixture with optimized conditions for [^{11}C]**4**. Retention time for reference standard of phenylacrylamide (**4**) = 5.55 min.

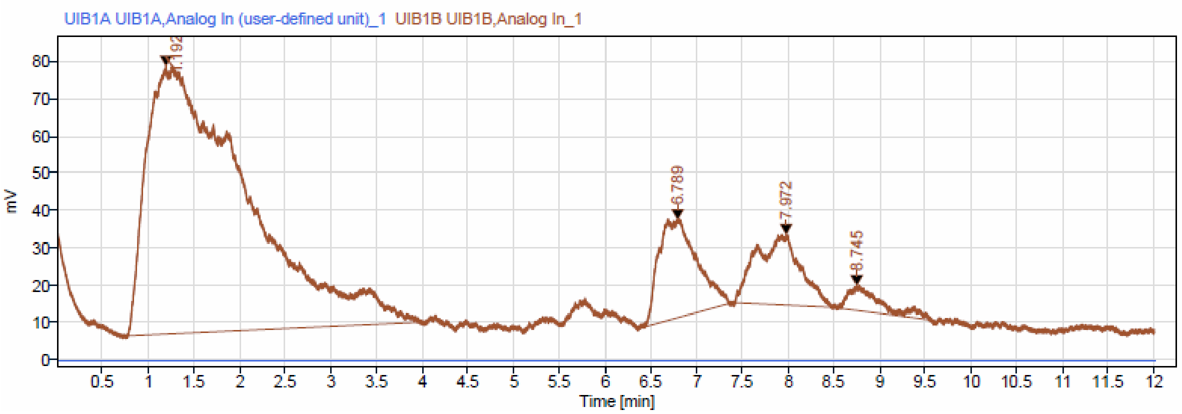

Figure S7. Radio-HPLC chromatogram of reaction mixture with optimized conditions for [^{11}C]**5**. Retention time for reference standard of diphenyl urea (**5**) = 6.55 min.

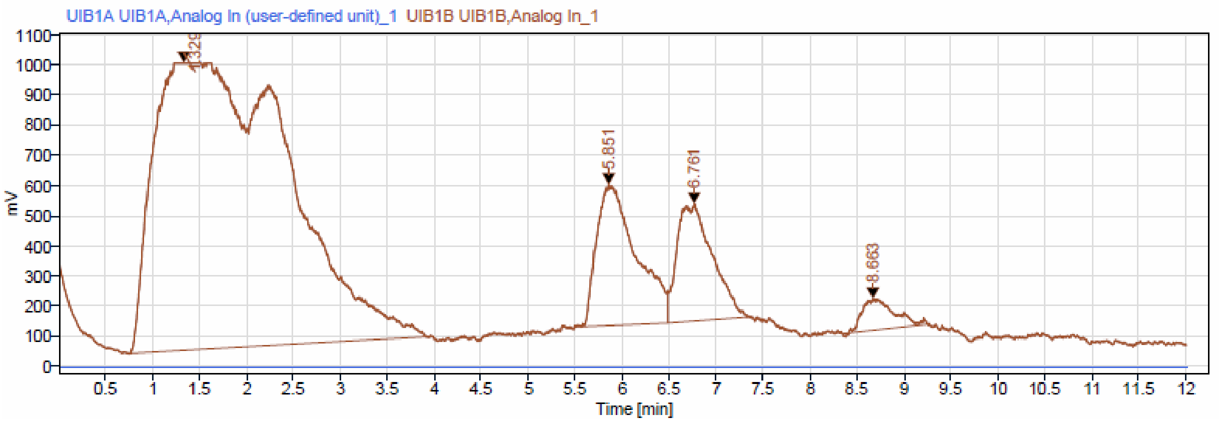

Figure S8. Radio-HPLC chromatogram of reaction mixture with optimized conditions for [^{11}C]**6**. Retention time for reference standard of benzyloxazolidinone (**6**) = 5.65 min.

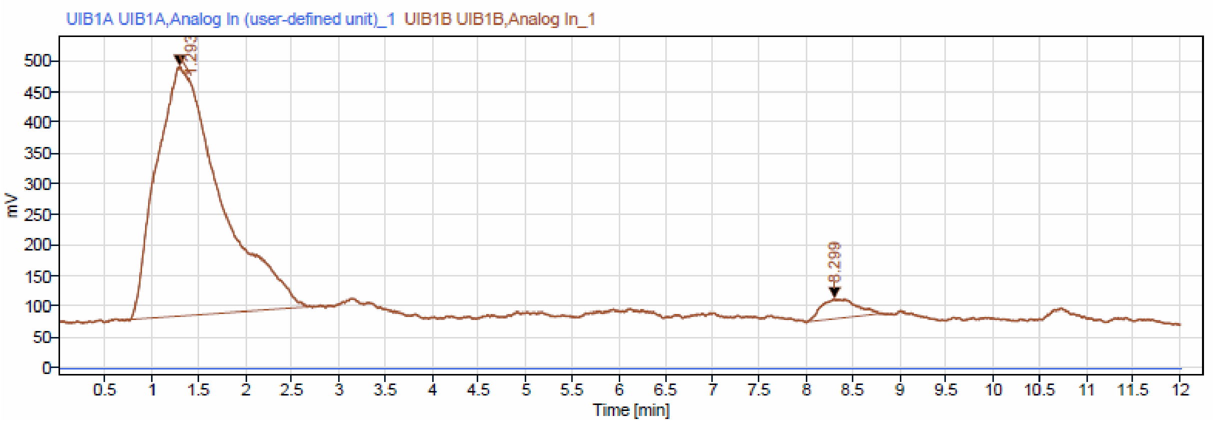

Figure S9. Radio-HPLC chromatogram of reaction mixture with optimized conditions for [^{11}C]SL25.1188. Retention time for reference standard of SL25.1188 = 8.25 min.

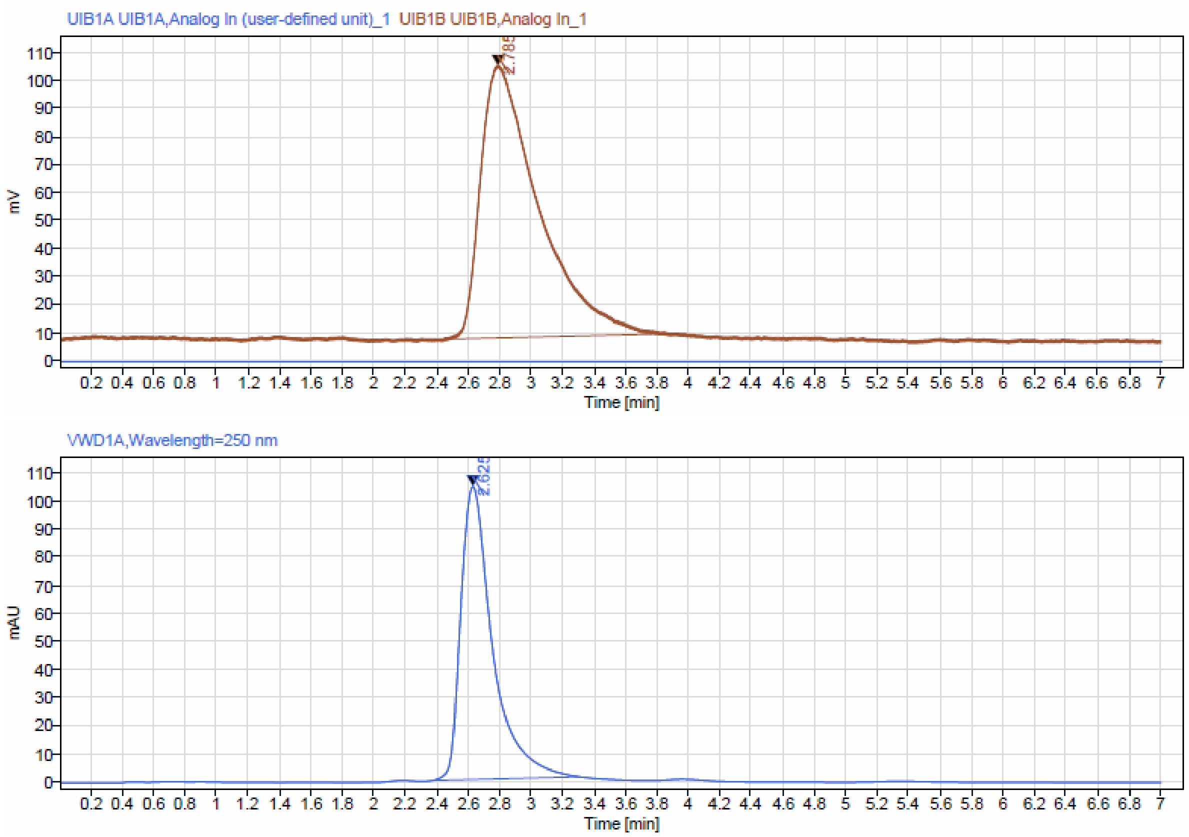

Figure S10. Radio-HPLC chromatogram of isolated [^{11}C]evobrutinib co-injected with unlabeled evobrutinib. Retention time of evobrutinib = 2.6 min.

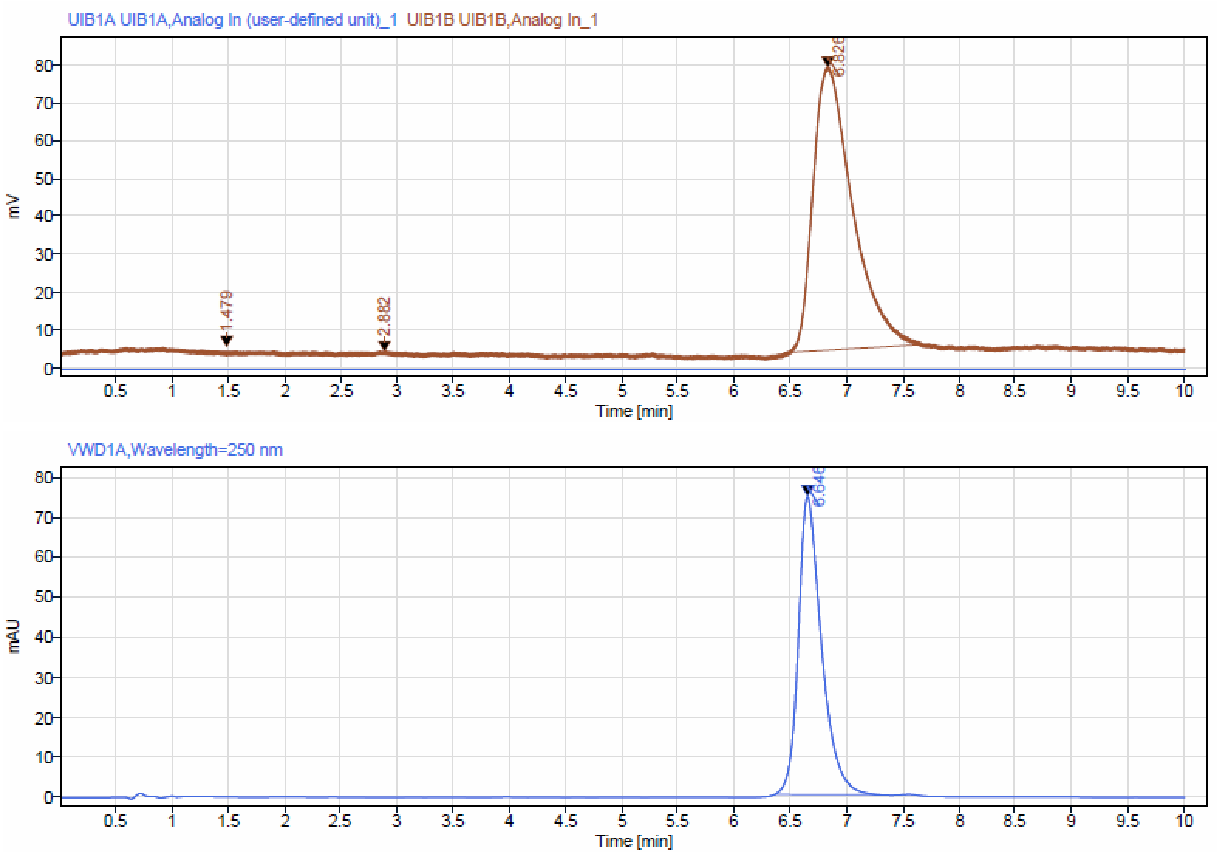

Figure S11. Radio-HPLC chromatogram of isolated [^{11}C]ibrutinib co-injected with unlabeled ibrutinib. Retention time of ibrutinib = 6.6 min
